# Understanding clinician perspectives on antibiotic associated adverse events to inform feedback

**DOI:** 10.1017/ash.2023.265

**Published:** 2023-09-29

**Authors:** Jerald Cherian, George Jones, Taylor Helsel, Zunaira Virk, Alejandra Salinas, Suzanne Grieb, Sara Keller, Pranita Tamma, Sara Cosgrove

## Abstract

**Background:** Feedback regarding antibiotic-associated adverse events (ABX-AEs) may assist clinicians with antibiotic decision making. We sought to understand how clinicians account for ABX-AEs when prescribing and their preferences for ABX-AE feedback. **Methods:** We conducted 1-hour virtual focus groups with 3–5 physicians or advance practice practitioners (APPs) per session at Johns Hopkins Hospital. Participants discussed the role of ABX-AEs in antibiotic decision making and feedback preferences. Participants evaluated prespecified categorization (mildly, moderately, or very concerning) of several ABX-AEs. Focus groups were recorded and transcribed. Transcripts were coded inductively by 2 independent reviewers; discrepancies were resolved by consensus. Codes were used to conduct thematic analysis. **Results:** Overall, 3 focus groups were conducted with 12 participants: 41.6% were house staff, 16.7% were attending physicians, and 41.6% were APPs. Most were female (91.6%) and were white (41.7%) or Asian (41.7%). Clinicians generally agreed with the prespecified categorizations of ABX-AEs based on degree of clinical concern (Table). We identified 5 themes: (1) The risk of ABX-AE is considered during initial prescribing but influences agent selection more than the decision to prescribe antibiotics. (2) The occurrence of an ABX-AE leads to assessment of need for continued antibiotic therapy. (3) The impact of an ABX-AE on other management decisions is as important as the direct harm of the ABX-AE when assessing severity. (4) Feedback must be curated to prevent clinicians from being overwhelmed with data. (5) Clinicians will be more receptive to feedback regarding ABX-AEs if feedback is contextualized (Fig.). **Conclusions:** The themes identified and assessment of ABX-AEs of greatest clinical concern may help inform the development of effective ABX-AE feedback methods to improve antibiotic safety.

**Figure:**
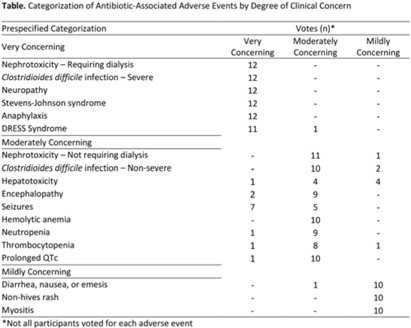

**Figure:**
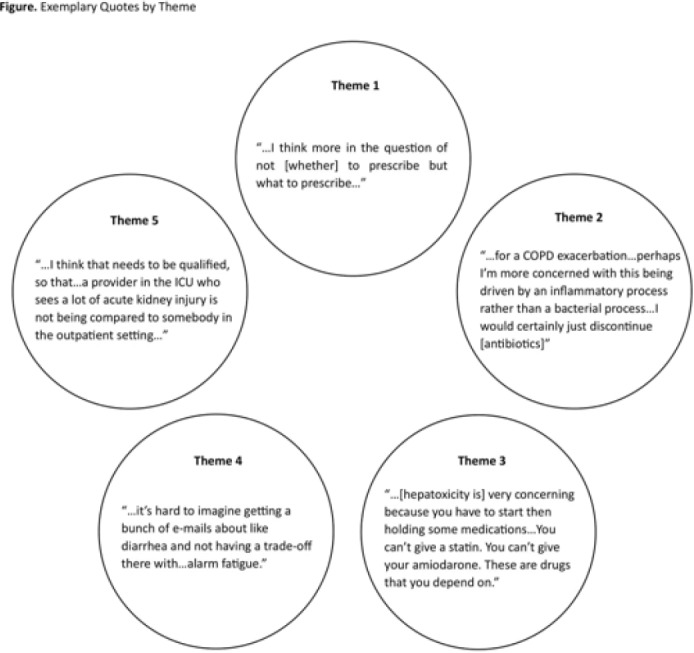

**Disclosures:** None

